# Beyond the “information deficit model” - understanding vaccine-hesitant attitudes of midwives in Austria: a qualitative study

**DOI:** 10.1186/s12889-021-11710-y

**Published:** 2021-09-14

**Authors:** Lisa Lehner, Janna Gribi, Kathryn Hoffmann, Katharina T. Paul, Ruth Kutalek

**Affiliations:** 1 Department of Social and Preventive Medicine, Center for Public Health, Medical University of Vienna, Vienna, Austria; 2 Present Address: Department of Science & Technology Studies, Cornell University, Ithaca, New York USA; 3 Konrad Lorenz Institute for Evolution and Cognition Research (KLI), Klosterneuburg, Austria; 4 Department of Political Science, Faculty of Social Sciences, University of Vienna, Vienna, Austria

**Keywords:** Vaccine hesitancy - MMR vaccinations - midwives - midwife training - health communication

## Abstract

**Background:**

Healthcare workers are considered key stakeholders in efforts to address vaccine hesitancy. Midwives’ influence in advising expectant parents on early-childhood vaccinations is unquestioned, yet they remain an understudied group. The literature on midwives’ attitudes towards vaccinations is also inconclusive. We therefore conducted an explorative qualitative study on midwives’ vaccine-hesitant attitudes towards MMR (measles-mumps-rubella) vaccinations in Austria.

**Methods:**

We conducted 12 in-depth interviews on their knowledge, concerns, and beliefs with midwives who self-identified as hesitant or resistant towards early-childhood MMR vaccinations. We analyzed the data using a grounded theory approach to distill common themes and meanings.

**Results:**

Healthcare workers’ stewardship to address vaccine hesitancy is commonly framed in terms of the “information deficit model”: disseminate the right information and remedy publics’ information deficits. Our findings suggest that this approach is too simplistic: Midwives’ professional self-understanding, their notions of “good care” and “good parenthood” inflect how they engage with vaccine information and how they address it to their clients. Midwives’ model of care prioritized good counseling rather than sharing scientific information in a “right the wrong”-manner. They saw themselves as critical consumers of that information and as promoting “empowered patients” who were free, and affluent enough, to make their own choices about vaccinations. In so doing, they also often promoted traditional notions of motherhood.

**Conclusions:**

Research shows that, for parents, vaccine decision-making builds on trust and dialogue with healthcare professionals and is more than a technical issue. In order to foster these interactions, understanding healthcare professionals’ means of engaging with information is key to understanding how they engage with their constituents. Healthcare workers are more than neutral resources; their daily praxis influenced by their professional standing in the healthcare system. Similarly, healthcare professionals’ views on vaccinations cannot be remedied with more information either. Building better and more diverse curricula for different groups of healthcare workers must attend to their respective roles, ethics of care, and professional beliefs. Taken together, better models for addressing vaccine hesitancy can only be developed by espousing a multi-faceted view of decision-making processes and interactions of healthcare workers with constituents.

## Introduction

In 2019, the World Health Organization identified vaccine hesitancy (VH) as one of the “10 Threats to Global Health” in light of increasing reluctance or refusal to vaccinate and a surge in cases of vaccine-preventable diseases [1]. This puts the study of VH on par with the research, development, and distribution of vaccines as core public health concerns [2, 3]. Within this field, uptake of vaccinations against measles disease is often cited as “the canary in the coal mine” for the overall state of national vaccination programs [4]. Though the vaccine is considered particularly effective [5], dropping numbers of combined MMR (measles, mumps, rubella)^1^ vaccinations highlight the negative shift perceived in public attitudes towards vaccines as a public health intervention [6]. This article focuses on a paradigmatic case of this trend: Austria, where vaccinations are voluntary and free, yet VH is comparatively high [7] and a total of 151 measles cases occurred in 2019 [8]. In recent years, especially rates for the second dose of measles-containing vaccine have dropped below 90% [9].

Research shows that healthcare workers (HCWs) play a key role in promoting vaccines [10–13]. They are seen as educators and “multipliers” of evidence-based information to remedy purported “information deficits” [14]. However, this “information deficit model” makes two related assumptions: (1) HCWs are “neutral” brokers of information who (2) must simply provide “more” information to constituents. Not only has the success of the information deficit model been variable in practice [15], this article questions the lack of attention hitherto paid to how exactly HCWs receive, digest, and propagate vaccination information. To do so, we focus on the exemplary case of Austrian midwives.

### The Austrian case

The issue of early-childhood MMR vaccinations is exemplary in Austria not only given rising incidence. Before the recommended age for MMR vaccines dropped to 10–12 months in the late 1990s, vaccinations were performed for school-aged children during school hours among a group of peers. The shift redistributed to (new) parents the responsibility to decide whether or not, and when, to vaccinate their children.^2^ This brings midwives’ position as ante- and perinatal counselors to the fore.

Generally claimed to harbor more hesitant attitudes towards vaccinations [17], midwives have overall received limited attention compared to other HCW groups in the context of international VH research [18, 19]. Their role in informing early parenthood and vaccine decision-making, however, has been made evident [20, 21]. In-depth analyses of certain groups and reasons for vaccine-hesitant attitudes promise instructive detail [22], yet existing research in Austria has used VH as catch-all explanation for critical attitudes [7] (Pichelstorfer A, Paul KT: Unpacking the role of metrics in global vaccination governance, forthcoming). Whereas studies generally focus on the attitudes of parents, in one study on the attitudes of Austrian HCWs midwives were conspicuously absent, hinting at their relatively marginalized role [23].^3^ Vaccinations are generally performed by physicians; childhood MMR vaccinations by pediatricians. Midwives’ role is largely limited to providing tailored advice, and they are not represented in governmental or advisory committees tasked with vaccination policy [25].

Austria is classically characterized a conservative welfare state with a universal, state-financed social health insurance (SHI) system and a “familistic orientation of its public policies” [26]. As such, maternity care is free of charge to all pregnant women, embedded in policies that guarantee extended maternity leave for up to 3 years with comparatively generous benefits. Maternity care is primarily hospital-based and overseen by physicians [27]. Obstetrician consultations during pregnancy are prerequisites for claiming public financial support, midwife consultations are not [28]. Midwifery care — if not during, then through home-visits for about 8 weeks after birth — is theoretically prescribed by law [29]. Systemically and in pure numbers, however, midwives remain in a niche position. Midwives do perform screenings, monitoring of vital signs, weighing, and blood tests, but are not the primary maternity providers as they might be in countries like Australia, the Netherlands, or the United Kingdom [30]. Their workload has been rightfully called “fragmented” in the past [31], yet this fragmentation crucially depends on the *kind* of midwife expectant parents can afford.

Though more complex in its details, the Austrian healthcare system can more simply be divided in a statutory public sector and a supplementary private sector. Midwives on duty in hospital maternity wards will attend births but generally do not maintain close and consistent contact with parents before or after birth. The majority of midwives, as represented in our study, work as independent midwives who accompany mothers during pregnancy, childbirth, and in the postnatal period. Their services have to be independently organized and paid out of pocket by expectant parents. Costs will vary depending on services but may lie upwards from €1500 or €2500 for home/extramural births.^4^ Parents can subsequently claim reimbursement from their public insurer, but only for 80% of the fee that would have been paid for the same service by a midwife with an SHI contact (tariff); non-tariff services are fully private.^5^ In contrast, services provided by SHI-contracted midwives are covered directly by public insurance at a lower general price-point. Such contracts are limited, however, and allocated by state, leading to geographic differences and shortages across Austria — around 17 such positions existed in Vienna at the time of the interviews [33]. Many midwives also work independently additional to upholding SHI contracts or being directly employed as attending midwives in hospitals.

Within this institutional pretext of the Austrian healthcare system, our article closely explores the specific concerns, beliefs, and attitudes of midwives who identify as hesitant or resistant towards early-childhood MMR vaccinations in their relation to expectant parents.

### The “information deficit model”

Social-science research has shown that publics’ hesitant attitudes towards vaccines are not so much the result of information deficits, nor reducible to a single choice, but rather embedded in a web of cultural attitudes, ideas, and health-related decision-making [34–37]. In similar ways, considering HCWs neutral information brokers falls short of such complexities. Different groups of HCWs may engage publics differently on issues of vaccination depending on professional ethics and roles [38]. Maes has shown that community HCWs’ views were dependent on their respective skills, perspectives, and desires; their motives and criticisms entangled in local histories, policies, and systems of power [39]. The pursuit of public health goals under the stewardship of HCWs can therefore benefit from a deeper understanding of these factors rather than considering HCWs mere “human resources” [40, 41].

In this vein, our article discusses results from a case study on Austrian midwives’ rejection or hesitancy towards MMR vaccinations. In their exemplary review of the literature, Attwell and colleagues [17] identified a broad spectrum of beliefs among midwives — ranging from generally favorable [42, 43] to considerably more vaccine-cautious than general practitioners or nurses [38, 44–46]. Noting this “paradoxical” range in midwives’ attitudes about childhood vaccinations, the authors urge more research. Most studies done recommend measures to *remedy* midwives’ information deficits regarding vaccines, rather than understanding their particular role in vaccination practices.^6^ Thus, the aim of this study was to address this gap by exploring the concerns, attitudes, and beliefs of midwives who identify as vaccine-hesitant or resistant to vaccinations. Specifically, it explores how midwives leverage their professional roles, ethics, and their ideas about “good parenthood” as catalysts to engage with vaccine information.

In the following, we first detail our methodology. We then present our findings, focusing on three particular themes that emerged in our inductive analysis: midwives’ role and self-understandings; the notion of care that shapes their practices and epistemic stances; and the links they draw between lifestyle choices and vaccination attitudes. We conclude by situating these findings in the current literature and formulate a set of recommendations as to moving beyond the current information deficit thinking.

## Methods

### Background

This research was conducted at the Medical University of Vienna as part of multiple qualitative studies supervised by one of the authors (RK) that investigated vaccine-hesitant attitudes of parents and HCWs towards childhood MMR vaccinations in Austria [(Ecker F, Kutalek R: “I’m not an anti-vaxer.” Vaccine hesitancy among physicians: a qualitative study, forthcoming)].

### Methodology

Qualitative studies are apt tools to explore topics hitherto under-researched and interviews allow exploration of reasons, meanings, and dogmas in what is said. The underlying constructivist paradigm holds that “the ‘reality’ we perceive is constructed by our social, cultural, historical and individual contexts” [48], so that the aim is not generalizability but in-depth understanding. The study was guided by the principles of Grounded Theory [49], used to inductively build theoretical conclusions from empirical data.

### Data collection

We recruited midwives who self-identified as hesitant or resistant towards MMR vaccinations and/or vaccinations in general, meaning that they claimed for themselves the label “vaccine skeptical” in the German vernacular by responding positively to our recruitment efforts.

Initial recruitment in/around Vienna using opportunity sampling via vaccine-hesitant physicians, on Facebook message boards, and at midwife centers or the National Committee of Midwives proved unsuccessful. Midwives often declined participation claiming that the topic was too contentious, and they were not knowledgeable enough or did not share their opinions with parents. Telephone calls (50), with contacts sourced from the public directory for midwives or through online searches only yielded three initial interviews. We then proceeded by snowball sampling and word-of-mouth from participants provided a majority of interviews. After eight interviews, JG actively sought out midwives to ensure a broader distribution of significant characteristics in the sample such as age, vaccination attitudes, and employment status (targeted sampling). Participants were recruited until no new relevant knowledge was obtained (data saturation).

Interviewees were between 28 and 58 years of age, all identified as women, and variably worked as independent, attending, or SHI-contracted midwives. A majority were working (also) as independent midwives and a minority had SHI contracts. This mirrored the overall employment distribution of the around 440 midwives employed in Vienna at that time [33, 50] (Table 1). The 12 interviewees covered the full spectrum of VH (Table 2).

**Table 1:** Breakdown of age and employment position of participants

Age Distribution		Employment Position	
20–29 years	1	Independent midwives (I_M)	6
30–19 years	2	Attending midwives (A_M)^a^	2
40–49 years	4	SHI-contracted midwives (SHI_M)^b^	4
50–59 years	5	Hospital-based midwives^c^	0
	**12**		**12**

^a^Both attending midwives also worked as independent midwives.

^b^Of the four midwives who upheld contracts directly with the SHI, three also worked as independent midwives.

^c^Hospital-based (attending) midwives who do not also work independently were not included in this study

**Table 2:** Breakdown of self-identification of participants towards (MMR) vaccination

Midwives’ self-identification^**d**^	… towards vaccination in general	… towards MMR vaccinations
**favorable**	0	3
**unsure**	7	4
**critical**	5	5

^d^All of the midwives we interviewed claimed a particular position on the topic of vaccinations, particularly MMR vaccinations, during the interviews. JG and LL coded midwives’ self-positioning independently from one another, eventually agreeing on the breakdown as noted in the table

We conducted the interviews as semi-structured, narrative one-on-one interviews between May and November 2017, either at home (5) or at midwives’ place of work (7). Interviews were 30–60 min long, conducted in German, audio-recorded, transcribed verbatim (JG), and subsequently translated for publication (LL). An interview guideline assured comparability between interviews and provided overall structure. Questions were first developed deductively from existing literature, but continually refined inductively in line with Grounded Theory principles to integrate initial findings. They covered three general areas: (1) professional position, training, and continuing education; (2) attitudes towards measles disease and MMR vaccination, information, consultation, and public perception in Austria; (3) personal experiences with vaccination practices. Interviews remained open-ended to allow interviewees to introduce their own topics, expound on their beliefs, and use their own words.

### Data analysis

LL analyzed full transcripts using Atlas.ti [51] and employing a coding approach based on Grounded Theory [52]. The purpose of a three-tier coding strategy was to build up thick, theoretical analysis in multiple rounds of inductive compression of the empirical data. For this article, a first round of open coding condensed the data thematically. As themes emerged across all interviews, LL used memos to assure transparency with regard to the developing analysis. During axial coding, similar codes and topics were compared, aggregating them into overarching themes with multiple respective characteristics, which encompassed: vaccine-hesitant/anti-vaxx attitudes and reasons; pro-vaxx attitudes and reasons; relationships to parents and other HCWs; midwifery practices and professional ethics; handling of and engagement with information; and general worldviews. In a last round of selective coding, these overarching themes were put in relation to one another (dependencies, overlaps, exclusions, conflicts, connections). This allowed us to develop a deeper understanding of how midwives’ vaccine-hesitant and/or resistant attitudes worked and how they made sense of their beliefs. Multiple rounds of feedback and discussion among all co-authors produced the final analysis presented below, which focuses on midwives’ engagement with vaccine information as inflected by their self-understanding, notions of care and ethics, and views on “good parenthood.”

### Ethical considerations

The study was approved by the ethics review board of the Medical University of Vienna. Interviewees were informed of the course and aims of the study beforehand, provided written and verbal informed consent on a pre-approved consent form, and could decline answering questions or discontinue the interview at any point, though none did. To protect midwives’ professional position they were guaranteed pseudonymity; all names used are pseudonyms and additional identifying information was redacted.

## Findings

### Midwives’ self-understanding

Interviewees considered themselves in a marginal role within the maternity care system in Austria with regard to vaccinations. They often expressed this view by pitting their professional identities against those of pediatricians and by articulating distinct ideas about their respective jurisdictions in vaccine decision-making. They put vaccinations within physicians’ purview as progenitors of Western biomedicine, in charge of performing and speaking authoritatively on vaccinations. Zoe, for instance, admitted that she was *“already a little too far distanced from measles vaccinations. It’s a topic to discuss with the pediatrician, very specifically. Because I think that is the person who kind of carries it all”* (P1, 20–29 years, I_M). Many of the midwives therefore talked about sending their clients to discuss vaccinations with their physicians.

Many of our interview partners described cooperation during maternity care despite potential professional divides. Some, if they had the chance, recommended pediatricians they trusted or “matched” pediatricians to the vaccination attitudes of parents as they came to know them. Rita talked about *“handing over*” (P3, 40–49, I_M) the issue of vaccination decision-making to a pediatrician with whom she closely cooperated. Yet, for Zoe and many of the others, such professional distance to vaccinations engendered tension. They talked about having to actively stop themselves or blocking parents from asking further questions. Hannah noted, *“Almost every third [woman] or every third couple asks me [about vaccinations] during postnatal care. And I don’t engage in that. I’m not the person to talk to about that”* (P11, 50–59, SHI_M).

Overall, the midwives described vaccinations as a *“hot-button issue*.” In their experience, vaccination decisions did not appear clear-cut but an urgent topic of discussion and involved lengthy deliberations for their clients before birth. Yet, in their view, lack of time and preparation posed significant hindrances in meeting these needs. An ideal process of awareness-raising about vaccinations would prepare expectant parents, especially mothers-to-be, well in advance. By this measure, many of the midwives found the system — for lack of patience or time — ill-equipped to address their clients’ questions and doubts. Our interviewees often saw a worrying disconnect between what they thought was the best care for expectant parents and what was provided by pediatricians. Stephanie voiced a common concern regarding interactions between parents/mothers and doctors, *“It’s a problem. Some mothers, they can’t find a pediatrician anymore who can respect a mother who says, ‘I don’t want to vaccinate my child”* (P2, 50–59, I_M). Midwives recounted stories of pediatricians refusing to see anyone who questioned vaccinations. Ida mused that pediatricians *“only hear, ‘I don’t want to vaccinate’ and— alarm, alarm! Because it’s already the 15th mother that day, who doesn’t want to vaccinate, and of course he [sic] might get impatient then*” (P4, 30–39, I_M). Indeed, some of the midwives doubted the capacity of overburdened state-funded healthcare to meet the need of comprehensive information and deliberation.

### Midwife model of care and information politics

The midwives did not see themselves as usurping physicians’ jurisdiction in propagating information about vaccinations. Hannah noted that midwives inhabited only *“a very narrow area of medicine”* (P11, 50–59, SHI_M). Though, within that area, they understood their practices as moored in professional ethics and a notion of “good care” distinct from physicians.

For those midwives we interviewed, *good* midwifery meant camaraderie with other midwives, sharing knowledge amongst one another, providing women in their care with thorough attention, and acting as knowledgeable counselors. In this, the midwives pitted themselves against an engagement with clients that, in their view, issued instructions and gave orders — a model of care they associated with (bio)medicine. Consequently, it was important to the midwives that they kept their distance from clients’ vaccine-decisions because they understood themselves neither as authorities nor *“missionaries”* of any single position. They appealed to taking a *“neutral”* stance, leaving final decisions to their clients.

Many felt that it was their professional responsibility to read up on vaccinations and recommend further information to clients. Indeed, all of the midwives interviewed detailed the many sources they accessed for information: books, message boards, the internet, and personal contacts among midwives or physicians. Asked how they had learned about vaccinations during their training, our interviewees recounted that it had hardly been part of their curriculum except for the national immunization plan. Patricia directly connected the plan to state governance, for whom she saw physicians acting as messengers, *“Physicians don’t give good explanations around vaccinations. They don’t know a lot of things, I feel like. Many don’t really have a plan and follow what the Ministry of Health or the National Board of Vaccination recommend”* (P5, 50–59, I_M).

Patricia expressed a view common to some of our interview partners: that vaccinations represented a *“regulation”* (P9, 40–49, I_M) from state authorities. They saw physicians as acting in a *“one-sided,”* pro-vaccination manner. In the name of “neutral explanations,” the midwives instead endorsed alternatives to that mainstream. For them, being hesitant to accept vaccinations was about fighting the status-quo from what they considered a position of opposition and promoting individual choice and agency. *“It’s like in politics*,” summarized Rita. “*There is one party that governs for many years, decades if you like. And there’s an opposition. And that opposition is really important so that the governing party can’t just do what it wants”* (P3, 40–49, I_M). In so doing, she also hinted at a sense of marginalization that midwives experienced in this particular context.

An experienced midwife, Rita also saw alternative approaches as having been *“important for us in maternal care, in child-rearing, in childcare. So that the system gets broken up a bit, gets a bit more flexible”* (P3, 40–49, I_M). Astrid similarly demanded flexibility. She talked at length about how she had *“become skeptical over time”* — both as a professional and regarding vaccinations — while becoming more involved with groups of midwives that *“dared to experiment”* (P10, 50–59, SHI_M) in the 70s. Drawing on this history of opposition against the status-quo and midwifery ethics of care, both of them demonstrated hesitancy to follow immunization plans by the letter or seeing physicians do so. Like them, many of the midwives we interviewed considered their vaccine-hesitant attitudes their own personal beliefs. At the same time, the midwives often split the difference between their hesitancy about vaccines and their professional roles by adopting an arguably “neutral” stance. Coupled with possibly inadequate critical training, this saw many of the midwives treating variable sources of information as equal in their counseling of clients.

### Midwives and “the empowered patient”

Maternity care in Austria is primarily hospital-based and overseen by physicians [27]. In light of this, interviewees often commented on common conflations of people who were vaccine-hesitant with people who chose midwives. They saw this exemplified in the archetype of the “home-birth,” as this will be the preferred model for a lot of expectant parents who choose all-inclusive independent midwifery care. Dora noted, *“If you ask a ‘home-birth midwife,’ she is probably going to have more women who don’t vaccinate at all”* (P7, 40–49, SHI_M). For Barbara, this stereotype had real-life consequences for the treatment of women. She felt that pediatricians were particularly skeptical towards women who had home-births, so much so that *“when the pediatrician sees that they had a home-birth, he [sic] will say, ‘Ah, so you won’t want to vaccinate either.’ More or less, they put them in a box, ‘Those who have-home births, they don’t like vaccinations.’”* Barbara could not agree with that sentiment; rejection of vaccinations was not at the heart of it. What mattered was information and acting on it, “*Our parents just want to get informed. Maybe more so than others”* (P8, 40–49, I_M).

The proprietary *“our parents*,” juxtaposed with generalized *“others,*” pointed to a particular type of client more common for the midwives we interviewed. The midwives implicitly or openly referred to this type of client as an *“empowered patient.”* This patient did their research, asked questions, and was independent in their choices. Our interviewees generally considered it essential to encourage parents to *“remain critical”* of taken-for-granted interventions like vaccinations. Adding a gendered valence to this need for agency, interviewees often referred solely to “mothers” and “women” in this context, even if they otherwise talked about “couples” and “parents.” Here, they considered it a professional responsibility to *“activate women to inform themselves”* (P6, 30–39, I_M).

Such activations, though, came with demands. Some of the midwives we interviewed, like Jessica, considered it a parent’s duty to get informed. They had to have competence, Lisa noted, to “*defend themselves, so that the opposite side knows, ‘Okay, they are well informed and they can’t be palmed off with some kind of instruction’”* (P9, 40–49, I_M). The empowered patient, then, was privileged and had the means to make such investments. Indeed, many of the independent midwives provided care for affluent clients. Stephanie explained that *“it’s not the average mother that I work with who can afford a midwife and who values a personal midwife. They have to pay for that. I’m not an SHI-contracted midwife who really works with a variety of people”* (P2, 50–59, I_M). In this context, many of the midwives identified a larger commitment in choosing a “no-vaxx” *lifestyle* or one of delayed vaccination.

For most of our interviewees, vaccinations presented a generalized intervention that lacked a person-centered perspective focused on child and family. It also presented an artificial manner to induce life-long immunity that would come about naturally through falling ill and recovering. Many of our interviewees considered diseases *“not just meaningless,”* but as Lea noted, *“Midwives generally don’t go at it with a pathological perspective on things but in principle assume that the individual is in a state of health”* (P6, 30–39, I_M). Measles disease was framed as a *“childhood disease”* and thus a “natural” immunological life-event. Zoe thought it *“simply important that you somehow go through [measles]”* (P12, 50–59, SHI_M) during childhood, and Astrid was certain that *“childhood diseases aren’t anything dramatic. Because childhood diseases are childhood diseases. We all went through them”* (P10, 50–59, I_M).

By the same token, interviewees deployed strong beliefs in the influence of environment on childhood immunity, especially familial units. A complex interplay of strengths and weaknesses had to be considered and actively provided for. Getting sick and being able to recover was mediated by multiple factors like whether or not children were breastfed, receiving an *“ideal”* diet, and living in a *“sheltered”* familial environment. Measles could be managed without vaccinations but that required guidance from trusted HCWs and demanded competence from parents, as well as emotional awareness. Lea cautioned that *“sitting there and watching”* your child, sick with measles, was *“a path that you need to be able to endure”* (P6, 30–39, I_M). Parents also needed time and the means to care for a sick child. Sick children were best kept at home for as long as they needed to*.* Ida was blunt about parents who did not enjoy the necessary socio-economic opportunities in that “*they really just have to vaccinate their children, simply because they don’t have the time to care for their really sick children”* (P4, 30–39, I_M). Vaccinations became important, therefore, for children who required *“out-of-home childcare”* in nurseries or kindergartens as they might not be *“as stable within or not as protected”* (P12, 50–59, SHI_M). In these considerations, affluence and/or traditional familial structures were implied: the economic means for parents to stay home for prolonged periods of time and a family model with one stay-at-home parent — more often than not, in the context of Austria’s maternity leave provisions, this meant mothers.

## Discussion

Our article provides an in-depth perspective on midwives’ concerns, attitudes, and beliefs regarding early-childhood MMR vaccinations. We especially highlight the shortcomings of the “information deficit model” on two fronts: First, by regarding HCWs as neutral brokers or “multipliers” of information, the model’s attendant logic affords little attention to the process of multiplication itself. Professional ethics around “good care,” the self-understanding of HCWs, and their role within the healthcare system inflect how they address their constituents and how they engage with information. Secondly, training for HCWs needs to take these aspects into account; simply providing *more* information to them will prove insufficient.

The midwives we interviewed situated themselves within the larger Austrian maternity care system by engaging in “boundary work” [53] against the professional jurisdiction of pediatricians. They framed physicians’ approach as technical, pointing to time constraints in the public SHI system, and leveraged cautionary tales of physicians refusing vaccine-hesitant parents. Such tales add meaning to midwives' consciousness of providing a “midwifery model of care” [17], distinct from that of “medicalization” [54] promoted by physicians. Though such dichotomization has been called into question in favor of focusing on “good maternity care” [55], as a discursive trope it helps us understand midwives’ professional ethics of care as it relates to vaccinations.

The midwives prioritized “naturalness,” the premise of physiological health, and the mother-and-child dyad. Within this frame, the midwives did not consider the science of vaccinations “theirs,” placing explanations to parents in the purview of pediatricians. Rather than sharing information in a “right the wrong”-manner, good midwifery care for our interviewees meant recommending further information, sharing personal opinion, or offering advice when prompted. Pointing parents to immunization guidelines, which they often assumed to be their designated role in the system, appeared insufficient to many and incompatible with being “good counselors.” Sobo, who has re-theorized vaccine refusal as an affiliative practice, notes that the act of refusal is “neither the primary feature nor the first step in refusing.” Rather, it is preceded by a selection process and a different understanding of the world and how its pieces hang together [56]. Many of the midwives saw in their opposition to vaccinations a source of agency, drawing variably on their ethics of care, the history of their profession as opposing medicalized gynecology [57], or a self-understanding as a critical “consumer.” This inflected how midwives received, engaged with, and promoted vaccine information, and had them assign equal value to a large variety of information. Whereas some public health commentators might see an information deficit — both in parents’ and in midwives’ attitudes of VH — a more complex picture emerges in our study: The midwives we interviewed saw a deficit only in that information hidden by “blind trust” [58] had to be countered with more diverse and arguably “neutral” information. The value of this information was judged in light of it proving skeptical of the status-quo in similar ways — and not soley or necessarily in light of its scientific character. Just like that, the purported deficit turns into a deluge. Like “restless consumers” [59], our interviewees felt an urge to pay “constant attention to [their] own choices,” saw themselves unable to “afford to be passive,” and did not show a lack but a restless pre-occupation with information procurement.

At the heart of this was midwives’ desire to remain and retain their clients’ capacities as “empowered patients,” just as they often saw themselves as empowered by virtue of their professional training. Only such could their clients evolve expertise and make independent informed decisions for their children in a nexus of neoliberal consumer culture, the democratization of expertise, and attendant demands on “good” parenthood [60]. For our interviewees, demands on their clients’ agency and responsibility were conduits for “alternative” conceptions of disease and appeals to “naturalness.” Nature, to them, often proved a more logical, safer, and more trusted arbiter of knowledge than “traditional” authorities like state and science. In a sense, nature inherently “just is” while state and science serve and are external influences. Paradoxically, while the heritage of second-wave liberal feminism and, therein, women’s inalienable rights to choose were palpable in the professional self-understanding of midwives [61], the logics of “naturalness” also reinforced traditional, (hetero-)normative conceptions of motherhood and familial arrangements. Midwives in our interviews highlighted the importance of mothers staying with their children for an extended period of time, refusing out-of-home care, and implied a “(male) breadwinner” model for the purposes of strengthening a child’s “natural” immune responses. Bobel has described this conjoining of choice and nature as “the paradox of natural mothering” and a particular instantiation of what it means to be a “good parent,” in which midwives and their clients appear to have become mutually implicated [62]. In this confluence, midwives especially placed their demands on women’s shoulders to actively exercise their rights to choose, stay informed, and secure their children’s health. Some of the midwives did so using their own experiences as mothers and choices in favor of “naturalness” as parameters. This view is reinforced in context, as Austria’s comprehensive maternity leave policies are disproportionately used by mothers and women disproportionately remain in part-time positions even as their children get older [63].

Yet, such choices remain unavailable to parents and women with the least resources. In that sense, midwives in Austria have become shorthand for a particular kind of middle-class parenting, linking affluence and VH [64, 65]. As noted above, the Austrian maternity care system requires substantial social and financial affordances from expectant parents. Few SHI-contracted positions, high demand with limited supply, fragmented care, and only a limited number of (home) visits fully covered by insurance before and following the birth place a lot of the onus on (expectant) parents to organize or “secure” midwife care independently. This ultimately creates a tiered system along class lines and its intersections with ethnicity and non-normative family types. In this system, affluence and socio-economic privilege translate into both opportunities and incentives to invest in choices: for personal midwives, individualized maternity care, and against vaccinations. This means being able to sustain a lifestyle where children can stay home with measles, time and effort can be expended on information gathering, and choices can be made that deviate from the norm and might cost parents financial benefits. As such, our findings on midwives’ practices and attitudes mirror those of studies on the decision-making logics of vaccine-hesitant or refusing parents [65–67]. More research on this particular dynamic in situ is outstanding, but in the case of Austria, it appears that the attitudes of (independent) midwives and their clients reinforce one another: Personal midwife services draw in affluent clients with an incentive and the means to invest in choices; conversely, these clients respond particularly to the midwifery model of care.

## Conclusions

The list of potential measures to address VH is long [68, 69]. Research in other countries has shown that midwives carry great responsibility for advising expectant parents on early-childhood vaccination decisions [20, 42]. In Austria, midwives’ role in matters of vaccination is fairly restricted, yet demands for midwives are expected to rise [50] and the success of midwife-led models of care in general obstetrics have been tried and proven [70]. Our study, exploring the perspective of practicing midwives in Austria, suggests that expectant parents do actively try to seek the advice of midwives they trust in the context of vaccination decisions, even in a system that tries to eschew midwives’ role in the matter.

In their paper on parents’ logics of vaccine hesitancy and refusal, Ward et al. respond to parents’ distrust of doctors and pharmaceutical companies — or, more generally, the larger system of Western biomedical authority — by posing a central question for public health going forward: “how do we get the message there in an appropriate manner and what message will be accepted?” [65] Our study suggests that midwives could be self-evident allies, but not ones who will perfunctorily act in a “right the wrong”-manner as implied by information deficit thinking. Enrolling midwives in any public health program addressed at parents’ vaccine decision-making must integrate a better understanding of how midwives engage with information and how they conceptualize “good care.” Providing more information to plug a purported deficit is not enough. A more critical approach requires addressing the larger systems in which these so-called deficits function, make sense, and are maintained.

Vaccine decision-making is more than a technical issue for parents, research shows. The evidence of science and medicine plays only a partial role. Similarly, the language of risks and statistics do not easily connect to the wellbeing of a single child and trust is built through dialogue [71–73]. Better conditions for engaging with VH cannot be fostered if we do not understand the interactions of HCWs and their clients. Our interviews made clear that midwives were deeply concerned with providing good care to their clients, but our findings track with international observations that midwives might not feel well-prepared on vaccination practices [74, 75]. In our view, lack of training regarding vaccination in the Austrian midwifery curriculum reinforces the notion that vaccination remains a doctor’s professional responsibility and competence. But better training, beyond the stipulations of immunization guidelines, will need to be better integrated into midwives’ overall ethics of care, address their understandings of disease, and critically engage with the larger worldview in which these are embedded. Only such, we believe, can better models for addressing VH be developed that espouse a multi-faceted view of decision-making processes, especially in the case of vaccinating children and protecting those, whose immunocompromised state does not give them a choice.

## Data Availability

The original audio data of the interviews generated during the current study are not publicly available to guarantee the pseudonymity of the study participants in accordance with the provisions of the ethics review board. Pseudonymized interview transcripts in original German are available from the corresponding author on reasonable request.

## Abbreviations

A_M: Attending Midwife
BMASGK: Bundesministerium für Arbeit, Soziales, Gesundheit und Konsumentenschutz (Austrian Ministry of Health, previous name)
BMSGPK: Bundesministerium für Arbeit, Soziales, Gesundheit, Pflege und Konsumentenschutz (Austrian Ministry of Health, current name)
CAM: Complementary and Alternative Medicine
HCW: Health Care Worker
I_M: Independent Midwife
MMR: Mumps, Measles, Rubella
RKI: Robert Koch Institute
SHI: Social Health Insurance
SHI_M: SHI-contracted Midwife
VH: Vaccine hesitancy
WHO: World Health Organization

## Footnotes

1 For clarity, we will proceed to refer to the vaccine as “MMR vaccine” since standalone measles vaccinations cannot be obtained. Colloquially and during interviews, however, we did consistently refer to “measles vaccinations.” Where the focus on measles requires it, we therefore still refer to measles disease “in the singular.”

2 Measles disease also dominates public and political discourse. A routine parliamentary inquiry in 2019 showed that the Austrian Ministry of Health had spent a total of about EUR 200.000 for “vaccination awareness” campaigns, with a majority of this money going to measles alone [16].

3 The closest comparison available is offered by a small-scale survey conducted by the Robert Koch-Institute (RKI) in Germany [24], which found that fewer than 70% of midwives within their sample supported vaccinating children and toddlers against Hepatitis B, MMR, and varicella; one fourth opposed measles vaccinations outright.

4 This estimate is based on the breakdown offered by the *Hebammenzentrum*, a private society for independent midwives, at https://www.hebammenzentrum.at/hebammen/kostenueberblick

5 A breakdown of tariffed services is available from the National Committee of Midwives (in German) at https://www.hebammen.at/eltern/kosten/, but the complex composition of covered versus private costs and services points to the high “search costs” characteristic of the Austrian system [32].

6 Recent studies have also cast doubt on a predominant assumption in the literature that simply links midwives’ vaccine-hesitant attitudes with adhering to “alternative” or “complementary medicine” (CAM) [47].
